# Crowdsourcing and machine learning approaches for extracting entities indicating potential foodborne outbreaks from social media

**DOI:** 10.1038/s41598-021-00766-w

**Published:** 2021-11-04

**Authors:** Dandan Tao, Dongyu Zhang, Ruofan Hu, Elke Rundensteiner, Hao Feng

**Affiliations:** 1 Department of Food Science and Human Nutrition, College of Agricultural, Consumer and Environmental Sciences, University of Illinois at Urbana-Champaign, 382F Agricultural Engineering Sciences Building, 1304 W. Pennsylvania Ave., Urbana, IL 61801 USA; 2 Data Science Program, Worcester Polytechnic Institute, Fuller Labs 135, 100 Institute Road, Worcester, MA 01609 USA; 3 Department of Computer Science, Worcester Polytechnic Institute, Worcester, USA

**Keywords:** Infectious-disease diagnostics, Bacterial infection, Risk factors, Information technology

## Abstract

Foodborne outbreaks are a serious but preventable threat to public health that often lead to illness, loss of life, significant economic loss, and the erosion of consumer confidence. Understanding how consumers respond when interacting with foods, as well as extracting information from posts on social media may provide new means of reducing the risks and curtailing the outbreaks. In recent years, Twitter has been employed as a new tool for identifying unreported foodborne illnesses. However, there is a huge gap between the identification of sporadic illnesses and the early detection of a potential outbreak. In this work, the dual-task BERTweet model was developed to identify unreported foodborne illnesses and extract foodborne-illness-related entities from Twitter. Unlike previous methods, our model leveraged the mutually beneficial relationships between the two tasks. The results showed that the F1-score of relevance prediction was 0.87, and the F1-score of entity extraction was 0.61. Key elements such as time, location, and food detected from sentences indicating foodborne illnesses were used to analyze potential foodborne outbreaks in massive historical tweets. A case study on tweets indicating foodborne illnesses showed that the discovered trend is consistent with the true outbreaks that occurred during the same period.

## Introduction

Foodborne diseases caused by the consumption of contaminated foods are an important public health issue that severely threatens the human health^1^. Approximately 4 million illnesses in Canada, 48 million in the United States, and 600 million worldwide, with 420,000 deaths, occur each year^2–4^. When two or more people become ill after consuming the same food, a foodborne outbreak occurs^5^. They usually result in significant losses in work time, economic burdens, and losses of precious lives^6^. In the United States alone, the annual economic cost of foodborne illnesses has been estimated to be $15–$60 billion, depending on the cost-of-illness model used^7,8^. The underreporting of foodborne illnesses is also an issue that affects the accurate estimation of the scale and economic burden of foodborne illnesses^9^.

Early detection of foodborne outbreaks would reduce the risk and curtail infections by means of product recalls and restaurant closures^10,11^. However, the current outbreak detection method in the United States, carried out by the Centers for Disease Control and Prevention (CDC), usually entails a significant delay between the first infections and when action is taken to inform the public about the incidence of an outbreak. In addition, risk assessment tools such as qualitative microbial risk assessment (QMRA) are based on assumptions and may not be able to facilitate a fast outbreak detection for reducing the losses^12^. On the other hand, these approaches are often relying on structured data that are collected via planned field-trial studies. These data are expensive to obtain and often not available in the most up-to-date form.

In recent years, the readily available and rapidly disseminated digital data (e.g., social media) have been utilized for detecting foodborne illnesses^13–18^. Crowdsourcing, a method that leverages massive online data from user responses, coupled with machine learning approaches, provide a new means for conducting food safety risk analysis and risk communications^19,20^. With crowdsourcing, labeled data can be obtained with low cost, which facilitates the preparation of training sets for building machine learning models^21^. Crowdsourcing and machine learning have been applied to the food safety field. For example, Ordun et al.^13^ used feeds from the open-source media outlets Twitter and Rich Site Summary to characterize the 2012 Salmonella event related to cantaloupes and estimate the numbers of sick, dead, and hospitalized. Harrison et al.^15^ developed a model to capture signals of foodborne illnesses from Twitter. Effland et al.^17^ employed Yelp reviews to build a system for detecting foodborne outbreaks from restaurants. The models have been adopted by a number of local health departments, including Chicago and New York City. Increasingly, the potential of employing social media data for public health surveillance has gained the attention of governments. In the past, prevention of foodborne outbreaks has mainly relied on reducing contaminations that can happen throughout the food supply chain, including the production, processing, packaging, transport, and storage^2^. In comparison, the incorporation of social media data explores the role that consumers can play in prevention of foodborne outbreaks.

Twitter (Twitter.com) has been recognized as one of the most popular social media platforms employed in public health-related studies^22^. Using text mining and machine learning techniques, researchers have explored the use of intelligent systems that can identify trending topics, mine consumer opinions, and capture food safety hazards from Twitter^23^. However, the characteristics of Twitter—short tweets, informal grammar, abbreviations, typographical errors, and hashtags—make the text analysis of the data challenging^24^. With the rapid development of natural language processing (NLP) technology, the state-of-the-art methods have improved the performances in various NLP tasks. The language model BERTweet, a variant of BERT (Bidirectional Encoder Representations from Transformers) was designed specifically for NLP tasks on Twitter^25^. The BERTweet model outperforms strong baselines in name-entity extraction and text classification tasks. While Twitter has been employed for detecting unreported foodborne illnesses, the previous studies have only considered a binary classification problem in which the models only predicted if a consumer’s post on social media indicated the occurrence of a foodborne illness, while failing to collect other critical information (e.g., food, location, and symptom) for predicting a potential foodborne outbreak^14–16^. Can the critical information related to foodborne illness incidences be extracted by the BERTweet model? To answer this question, this work aims to employ Twitter as the data source and modify the language model BERTweet to not only predict if a consumer’s post (a tweet) indicates an incidence of foodborne illness, but also to extract critical entities related to the foodborne illness incidence in an automatic manner. The key elements of time, location, and food will be detected in tweets related to the unreported foodborne illnesses and used as critical information for analysis of potential outbreaks.

Our major contributions are as follows:

- We collected high-quality crowdsourced data with multiple task labels for constructing models to detect potential foodborne illness cases.
- We effectively modified the state-of-art deep learning model BERTweet to our dataset and build a dual-task model that can both identifying cases and extracting important entities related to the cases, such as food, location, and symptom.
- We applied the model to a case study of lettuce outbreaks and captured some spikes from social media that are consistent with the patterns in the outbreaks.

## Related works

Social media platforms such as Twitter and Facebook are generating a rich amount of real-time text data for the analysis of human behaviors, sentiments, and trends^26^. The availability of these data also provide new opportunities to conduct surveillance of health matters^27^, such as influenza^28^, Ebola^29^, and the recent Covid-19 pandemic^30^. While foodborne diseases and food safety are significant public health issues, the utilization of social media platforms on food safety have been mainly focused on risk communication and consumer perception analysis instead of surveillance^31–33^. Only until recent years, scientists have developed machine learning models to automatically detect unreported foodborne illnesses from social media platforms, e.g. Twitter and Yelp^14–17^. In specific, Support Vector Machines (SVMs) is one of the most effective models with high accuracy in classifying the text data from social media^16,34^. However, previous models only focused on a coarse-grained inspection, i.e. building models that can identifying social media posts indicating foodborne illnesses, followed by a labor-intensive examination by experts. These approaches, though proven effective, is rather slow and costly.

The potential of big data and deep learning is attracting increasing attention in the food safety field^20^. In dealing with text data, advanced deep learning methods have been developed for specific type of data. Twitter has been used as a popular social media platform for public health topics^22^. Entity extraction helps to identify critical information such as location, event entities, and symptoms of significance to disease surveillance^35^. BERTweet, as a deep learning model specially designed for English tweets, has been proven effective in text classification and entity extraction tasks, which enables token-level (word-level) analysis of a tweet^25^. Combining token-level and sentence-level inputs significantly improved the performance of a convolutional neural network^36^. Annotations of entities such as drug names, diseases, and symptoms from Twitter data and relation analysis between the entities was effective in building classification models for pharmacovigilance topics^37^. Twitter was used to build models classifying foodborne illness incidences^14–17^. However, limited information is available predicting for new data using the models as they are only focused on sentence level analysis. Critical entities such as location, food, symptom that are related to the infected cases are missing, making it impossible to conduct pattern analysis or potentially outbreak prediction.

## Materials and methods

### Data collection

Twitter provides sampled data (~ 1%) for research purposes. In this study, both real-time and historical data were collected for enriching the dataset for analysis. Around 43 GB real-time tweets were collected through the Twitter Streaming API using a keyword filter starting from October 2020, which can download twitter messages in real time, Keywords were selected based on the relevance to foodborne illnesses including common symptoms and their variations, including ‘#foodpoisoning’, ‘#stomachache’, ‘food poison’, ‘food poisoning’, ‘stomach’, ‘vomit’, ‘puke’, ‘diarrhea’, and ‘the runs,’ while some very ambiguous words (e.g., ‘sick,’ ‘fever’) were discarded to exclude diseases not related to foodborne illnesses^17^. In addition, 36 GB historical Twitter data were collected through a third-party tool Twint from 2011/1/1 to 2020/12/31 with the same keyword list. The two datasets were mixed and sampled for labeling process.

### Crowdsourcing and human labeling

**Figure 1:**
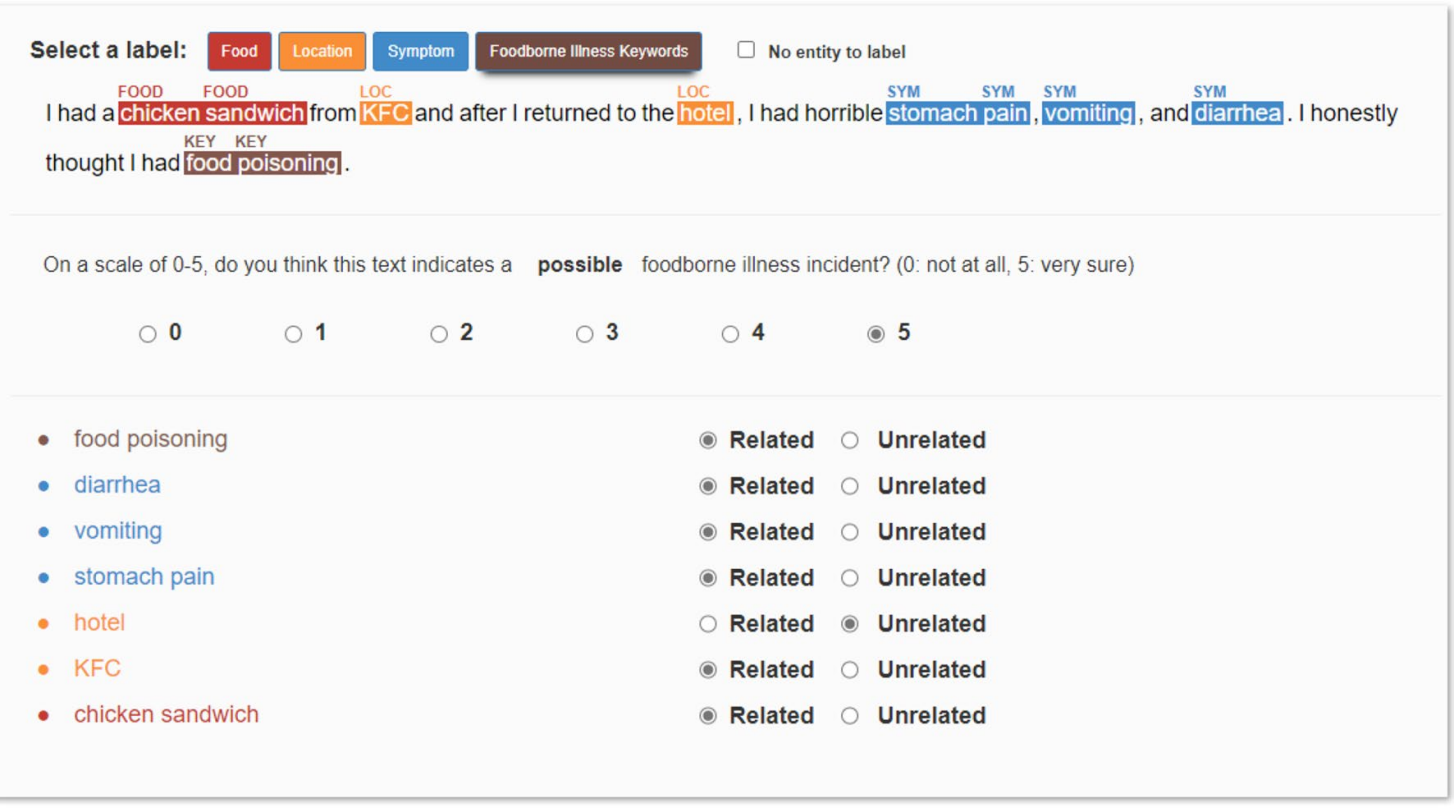
User interface used for data collection on Amazon Mechanical Turk.

Amazon Mechanical Turk was employed as the platform for crowdsourcing, in which registered labelers were recruited to complete the tasks. For a given tweet, workers were asked to read carefully, score on a scale of 0–5 on how much they agreed that the tweet indicated a possible foodborne illness incidence (0: not at all, 5: very sure), highlighted all words/phrases belonging to specific labels (food, location, symptom, and foodborne illness keywords), and decided if each of the highlighted words/phrases was related to the foodborne illness incidence. An illustration of the labeling interface is shown in Fig. 1.

**Figure 2:**
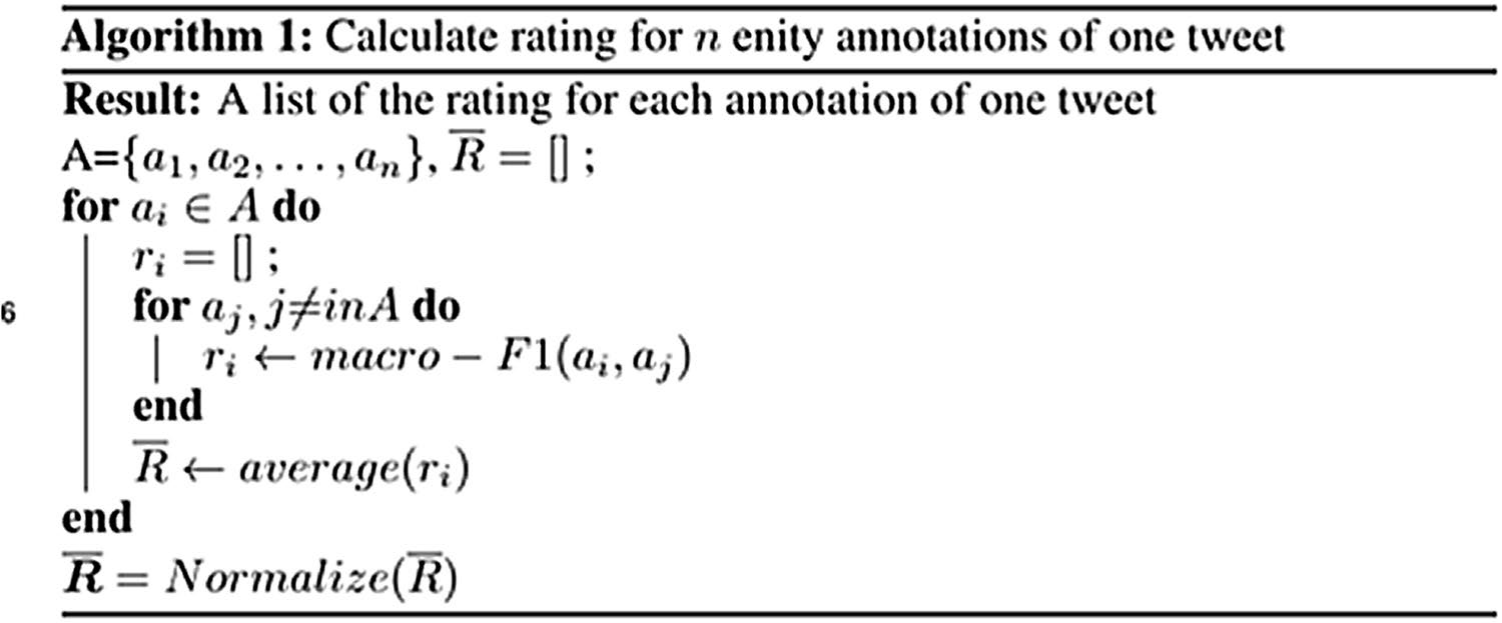
Pseudocode of the inter-worker agreement algorithm.

Three thousand tweets were selected from the pool mixed with historical and real-time data as the dataset to be labeled, and each tweet was assigned to five workers to collect the results. The quality of the labeled data is of significant importance to ensure model performance. Thus, a few steps were designed to identify and reject bad annotations to ensure data quality. First, the dataset was split into six batches and published on the Amazon Mechanical Turk platform one batch per time (i.e., 500 tweets/time). The maximum number of tweets that one worker can label was set to 10 in each batch to prevent spammers providing too many low-quality annotations. An algorithm modified from Finin et al.^38^ was adopted to evaluate the inter-worker agreement, for eliminating low-quality annotations. The pseudocode for the algorithm is shown in Fig. 2. The annotations with less similarity than the threshold (0.6) were rejected and republished for others to label to obtain high-quality annotations, while the spammers who labeled the low-quality annotations were blocked from labeling in future batches. By filtering out the low-quality annotations, the entity-level inter-worker agreement, expressed as Krippendorff’s alpha, was raised from 0.55 to 0.73. After collecting the labeled data, majority voting, a common method used in aggregating data from crowdsourcing, was used to aggregate the results from different labelers.

### Dual-task BERTweet model

**Figure 3:**
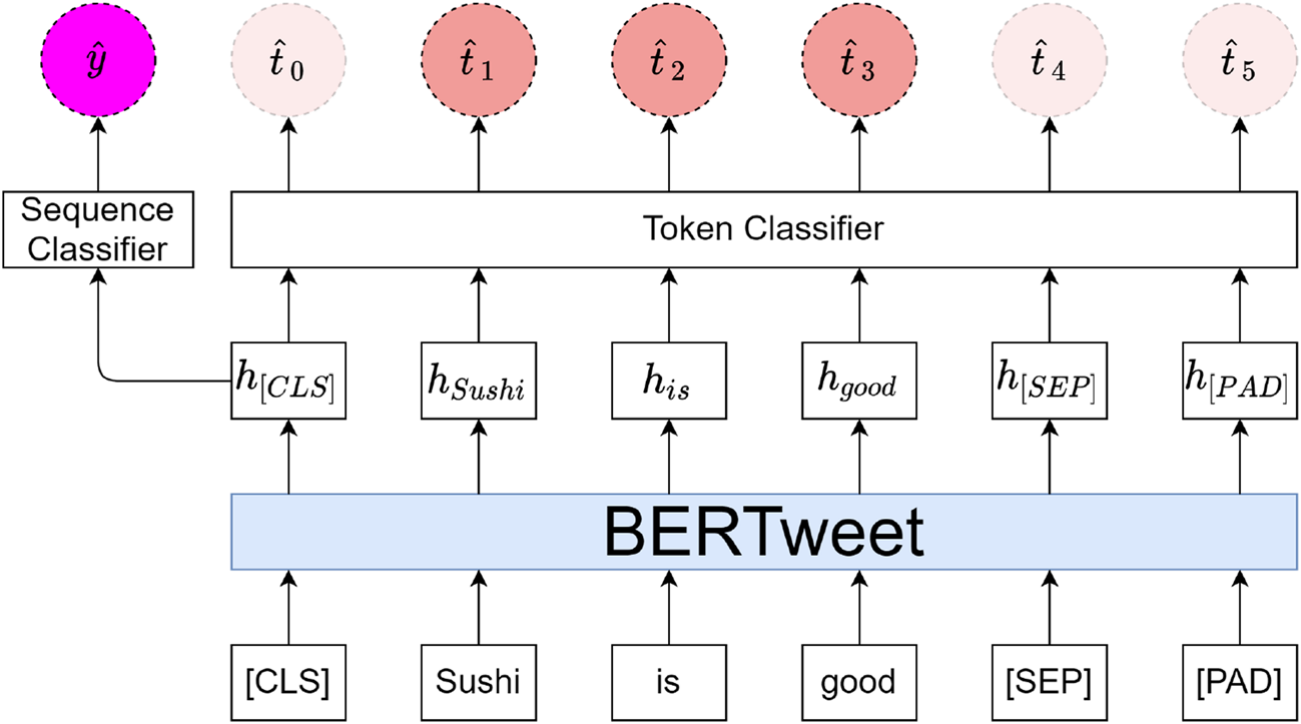
Diagram of the dual-task BERTweet model.

BERTweet is the first public large scale pre-trained language model for English Tweets. The model architecture of BERTweet is the same as the BERT base model. BERTweet uses the RoBERTa pre-training procedure. Experiments illustrated that the BERTweet outperforms state-of-the-art models on named-entity recognition and text classification tasks^25^. In this study, we aimed to design a language model that allowed us to complete two tasks, i.e., (1) to classify if a sentence/tweet indicated a foodborne illness incidence and (2) to extract entities related to the incidence, simultaneously. Therefore, a dual-task BERTweet model was developed by modifying the architecture of the BERTweet model, as shown in Fig. 3. Before feeding into the model, tweets were first tokenized and special tokens were added to the tweets. [CLS] was a special symbol added in front of every input sentence. [SEP] was a special separator added right after the end of every input sentence. [PAD] was added after the [SEP] token to make all input sentences have the same length. Then the model took the preprocessed tweet as the input. Then the BERTweet model generated a sequence of vectors that served as the representation of each token in the tweet. The representation of [CLS], having been pre-trained as the classification representation of input sentence, was fed into a sequence classifier to predict the relevance of the input tweet. Meanwhile, all token representations were fed into the token classifier to generate the prediction of the entity type of each corresponding token. The entity extraction and sequence classification can benefit each other by sharing the representations from the BERTweet model.

### Model training

The aggregated results of the 3000 labeled data were collected for machine learning with training, validation and testing, in which training set (2400 tweets) was used to learn a dual-task model best describes the dataset, validation set (300 tweets) was used to generalize the model, and testing set (300 tweets) was used to evaluate the performance of the trained model (see Supplementary Figure S1). The trained dataset was fed into the dual-task BERTweet model, which generalized when the validation set was introduced. The performance of the model was evaluated based on applying the trained model to the test set.

### Evaluation

Given a dataset, a binary classification gives a number of positives (Yes) and the number of the negatives (No). To evaluate a classifier, one needs to compare the predicted conditions with true conditions. As shown in Supplementary Table S3, the four numbers (true positive/TP, false negative/FN, false positive/FP, and true negative/TN) are the basics for computing performance metrics in binary classification tasks. The performance of each classifier was evaluated on the 300 tweets in the test dataset. For the task of sentence classification, we evaluated the performance of the dual-task BERTweet model in the sentence classification task using 4 common performance metrics: *precision*, *recall*, *F1-score* and *accuracy*^39^. *Precision*, or positive predictive value, is the proportion of true positives out of the total number of positive predictions, expressed as $\text{Precision}=\frac{\text{TP}}{\text{TP}+\text{FP}}$. *Recall*, or sensitivity, is the true positive rate, expressed by $\text{Recall}=\frac{\text{TP}}{\text{TP}+\text{FN}}$. *F1-score* is the harmonic mean of precision and recall, expressed as *F1-score* $=2\times \frac{\text{Precision}\times \text{Recall}}{\text{Precision}+\text{Recall}}=\frac{2\text{TP}}{2\text{TP}+\text{FP}+\text{FN}}$. *Accuracy* is the rate of predicting right out of all the predictions, expressed as $\text{Accuracy}=\frac{\text{TP}+\text{TN}}{\text{TP}+\text{TN}+\text{FP}+\text{FN}}$. The range for all the four metrics is between 0 and 1, with 0 being the worst score and 1 the best score. Among them, F1-score is most popular in evaluating binary classification problems, and will be adopted as the primary metric in result discussions.

We evaluated the performance of model on entity extraction task using the same four metrics in different expressions ($Precision = \frac{number\, of\, predicted \,entities}{{total\, number \,of \,predicted\, entities}}$, $Recall = \frac{number\, of \,predicted \,correct\, entities}{{total\, number\, of\, labeled \,entities}}$, $Accuracy = \frac{number\, of \,elements\, predicted}{{total \,number \,of\, elements}} , and$ *F1-score* = $2 \times \frac{Accuracy \times Recall}{{Accuracy + Recall}}).$ F1-score is also a popular metric used in evaluating entity extraction, thus will be mainly adopted in result discussions^40^.

### Frequency analysis

The dual-task BERTweet model was applied to the historical Twitter data collected from the 1/1/2018 to 12/31/2018. Given a tweet, the model gives two results—one is “Yes” or “No” for sentence classification task to classify if the tweet indicates a foodborne illness incidence, and another is sequence annotations for each element in that tweet, including predicted entities. For example, one tweet is “I got food poisoning from a grilled cheese last night and I’ve never felt so betrayed in my life.” The results based on the model are [“Yes”], [‘O’, ‘O’, ‘B-other’, ‘I-other’, ‘O’, ‘O’, ‘B-food’, ‘I-food’, ‘O’, ‘O’, ‘O’, ‘O’, ‘O’, ‘O’, ‘O’, ‘O’, ‘O’, ‘O’, ‘O’]. The non-O continuous tag sequence of the same type is an entity. Here, the model predicts two entities—one is “food poisoning” labeled as KEY entity, another is “grilled cheese” labeled as FOOD entity. Only when the sentence classification result was “Yes” and the FOOD entity was predicted, the tweet was included in frequency analysis, in which the changes in total number of relevant tweets with time were observed.

## Results and discussions

### Observations from a shallow data-processing pipeline

To obtain instant and historical online data related to foodborne illnesses, we first developed a data-processing pipeline that automatically extracted and detected information on potential food-poisoning incidents from Twitter posts. Tweets mentioning the keyword “food poisoning” in the timespan of January 1, 2018, to September 18, 2018, were extracted as potential signals of foodborne illnesses. The daily number of collected tweets as signal intensities was observed (see Supplementary Figure S2). The black line represents the raw data, and the red line presents the data after pre-processing (e.g., the removal of retweets and hyperlinks). Though the average intensity significantly declined after the preprocessing, the temporal pattern remained consistent. The peaks appeared in the months of March, April, June, and September, those related to the recent foodborne outbreaks. These results encouraged us to look closer at the content of each data point to figure out the potential correlations of these tweets to the outbreaks. Personal-experience tweets, which tend to use more personal pronouns (e.g., I, you, we), and also to have stronger sentiments (mostly negative), than those that are non-personal experience tweets (see Supplementary Table S4). Some entities identified by human taggers were listed in the example tweets, including symptoms common in produce-induced foodborne illnesses, and name entries of produce or food products (e.g., lettuce, salad, sandwich). However, this method is limited to the data collection mechanism with only one keyword (“food poisoning”). Harris et al. (2014) used the same strategy to collect data for building a machine learning model to classify foodborne illnesses from Twitter^13^. Effland et al.^17^ expanded the keyword list by including hashtags (e.g., “#foodpoisoning”) and other forms (e.g., “food poison”) of keyword “food poisoning,” and symptoms of foodborne illnesses (e.g., “stomach,” “vomit,” “puke,” “diarrhea,” and “the runs”). It needs to be clear that adding more keywords to the search strategy will collect more data potentially related to a foodborne illness incidence, while introducing more noises (irrelevant information) at the same time. In this approach, the method from Effland et al.^17^ was adopted for maximizing relevant dataset. On the other hand, the cost of identifying entities was rather high as it relied on human tagging, in which a machine learning approach would be more efficient by allowing the computer to detect the entities in an automatic manner. A dual-task BERTweet machine learning model was, therefore, developed to not only classify the unreported foodborne illness incidence, but also detect important entities related to the incidence.

### Performance of the dual-task BERTweet model

The performance of the dual-task BERTweet model is shown in Table 1. The F1-score, defined as the harmonic mean of precision and recall, is widely used as a popular metric to evaluate the performance of machine learning models^17,41^. The model achieved F1-scores of 0.87 and 0.61 on the sentence classification task and the entity extraction task, respectively. Of the 300 tweets in the test dataset, there are 203 positive examples (labeled as “Yes) and 97 negative examples (labeled as “No”) for the sentence classification task. In the protocol designed for human taggers on Amazon Mechanical Turk (see Fig. 1), the task of classifying if a tweet is relevant (“Yes”) or irrelevant (“No”) to a foodborne illness incidence was designed as point scale (0–5) instead of binary scale (“Yes” or “No”). It was based on the assumption that the point scale would help human taggers to make the decision easier when faced with ambiguous tweets. The results obtained were then transferred to a binary scale by denoting tweets labeled with 0–2 as negatives (“No”) and tweets labeled with 3–5 as positives (“Yes”) before feeding into the model. Some examples of tweets in the test dataset and correct and incorrect predictions are shown in Table 2. Many of the false positives cannot be identified by the model based on n-grams up to n = 3. For example, a tweet wrote, “It happens too fast to be food poisoning,” which would require 6-g for the model to capture the negation (too… to…). It illustrates a major shortcoming of language models based on n-grams: important relationships between words often span large distances across a sentence. Another major source of false positives are tweets that, though talking about an experience of food poisoning, either happened in the past or in a hypothetical/future sense, thus are not labeled as “Yes.” Similar observations were found in the study by Effland et al.^17^ on the task of classifying “sick” reviews from Yelp.

**Table 1:** Performance evaluation of the dual-task BERTweet model.

	Precision	Recall	F1-score	Accuracy
Sentence classification	0.8495 ± 0.0331	0.8867 ± 0.0343	0.8667 ± 0.0033	0.8153 ± 0.0102
Entity extraction	0.4927 ± 0.0343	0.8143 ± 0.0077	0.6134 ± 0.0271	0.9241 ± 0.0090

**Table 2:** Examples of tweets and predictions using the dual-task BERTweet model.

No	Tweets	Sentence label	Sentence classification	Entity label	Entity extraction
1	“It happens too fast to be food poisoning. It’s like I forced myself to eat too much, even though I didn’t eat that much and I was starving before.”	Not sick	Sick ($\times$)	[KEY]: “food poisoning”	[KEY]: “food poisoning, ” [SYM]: “starving”
2	“Never had one. Never will. I remember a number of years ago reading an article by doctor, who said a high percentage of supposedly stomach flu cases were actually food poisoning.”	Not sick	Not sick ($\surd$)	[KEY]: “food poisoning,” [KEY]: “stomach flu”	[KEY]: “food poisoning, ” [KEY]: “stomach flu”
3	“I got food poisoning from a grilled cheese last night and I’ve never felt so betrayed in my life.”	Sick	Sick ($\surd$)	[KEY]: “food poisoning,” [FOOD]: “grilled cheese”	[KEY]: “food poisoning,” [FOOD]: “grilled cheese”
4	“Text U know what’s said? I’m so OCD about washing my hands and not getting sick, everyone around me doesn’t care and guess who gets food poisoning.”	Sick	Not sick ($\times$)	[KEY]: “food poisoning”	[KEY]: “food poisoning, ” [SYM]: “sick”

$\times$ means incorrect predictions and $\surd$ means correct predictions.

The performance of the dual-task BERTweet model on sentence classification task was based on the number of sentences predicted by the model. In contrast, the performance of the model on entity extraction task was based on the number of entities predicted by the model. Some examples of entity extraction are shown in Table 2. It can be seen that all the labeled entities were predicted correctly by the dual-task BERTweet model, while the model also predicted some entities that were not labeled. For instance, words such as “starving” and “sick” were predicted as symptoms (simplified as SYM) while they were not labeled in the dataset. Keywords (simplified as KEY) such as “food poisoning,” or food names (simplified as FOOD) such as “grilled cheese,” labeled out in the dataset, were also predicted correctly by the model. It indicates that the dual-task BERTweet model is capable of capturing most of the important entities related to a foodborne illness incidence. It allows us to collect critical information such as food, symptoms, location, which would be impossible with human tagging when the dataset is large as the Twitter streaming data. Along with the time information provided by the collected dataset, one can conduct event detection of potential foodborne outbreaks by utilizing the collective data.

**Figure 4:**
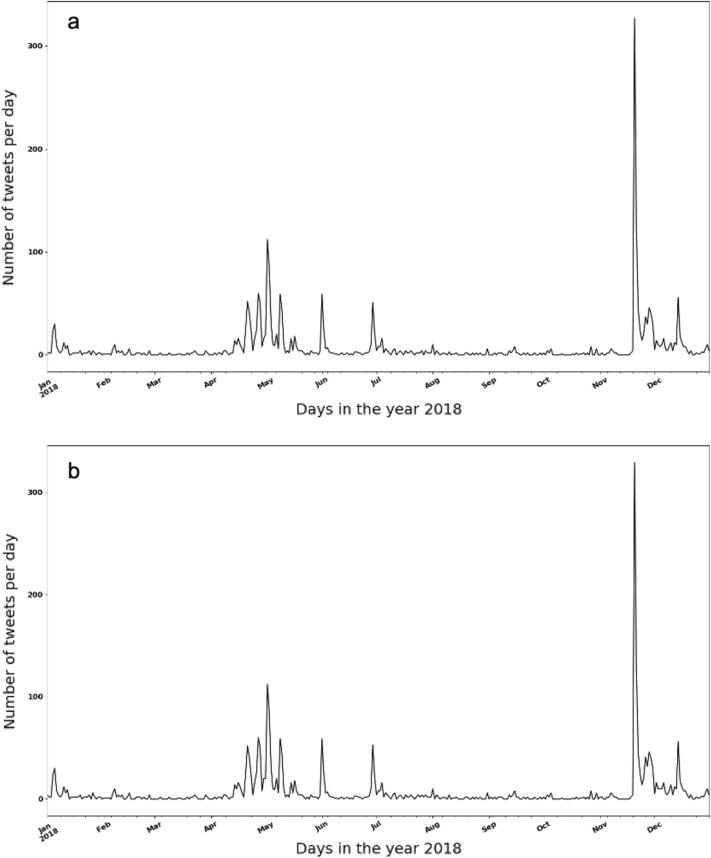
Number of tweets related to foodborne illness incidents associated with lettuce (**a** extracted by the dual-task BERTweet model and **b** extracted by keyword searching).

The changes in the number of tweets indicating foodborne illnesses associated with lettuce without location differentiation is shown in Fig. 4, in which Fig. 4a is the result extracted by our dual-task BERTweet model, (food entity = “lettuce”) and Fig. 4b is the result extracted by keyword search (keyword = “lettuce”). It can be seen that the two results look almost the same, with similar spike distributions across time, evidencing the high precision of the dual-task BERTweet model in extracting specific entities. While keyword searching is a common method to extract relevant information for a given food item, our model has the advantage that it can extract all food items automatically without supervision. In reality, the food item causing a foodborne outbreak is often unknown during initial period or even till the end of the events. Therefore, this model provides an opportunity to monitor the changes of potential risky food items with time for assisting outbreak investigation.

Social media has been utilized to detect unreported foodborne illness incidences in a number of recent studies, and the methods were adopted by several local health departments, including those in Chicago^14^, Las Vegas^16^, New York City^17^, and St. Louis^42^. The models developed by these works are summarized in Supplementary Table S5, in which the name of the models and their performance in predicting Twitter data using F1-scores are listed. These models were used as baseline models for comparing with our BERTweet model. In the individual models that only classify foodborne illness incidences, the F1-scores of the Foodborne Chicago model and of the FoodSafety SLT model were not provided in their reports, while the F1-score of the FINDER model was 0.74. The dual-task model developed by Effland et al.^17^ was recognized as the one with best performance in the task of classifying unreported foodborne illness incidents from social media. In contrast with our study, the dataset they used was Yelp reviews instead of Twitter posts. The F1-score for the task of classifying “sick” reviews was 0.87 as indicated in their paper. By deploying the FoodborneNYC model released on Github.com to analyze tweets, we found that the F1-score dropped to 0.84, which could be attributed to the differences in the two datasets. The F1-score of our dual-task BERTweet model is 0.87, meaning that, in the task of classifying unreported foodborne illness incidences from Twitter data, our model outperforms the previous models. In addition, this model also can extract entities of importance to conduct outbreak analysis. The FoodborneNYC is also a dual-task model that can classify if a review contains “multiple” illnesses, which might be indicative of a potential outbreak from one restaurant. However, large-scale outbreaks such as multistate outbreaks are not occurring in one restaurant. The advantage of using entities related to foodborne illness incidences is that it is not restricted by location, and thus has potential to be applied to detect large-scale outbreaks.

### Trends of foodborne illnesses across time

**Figure 5:**
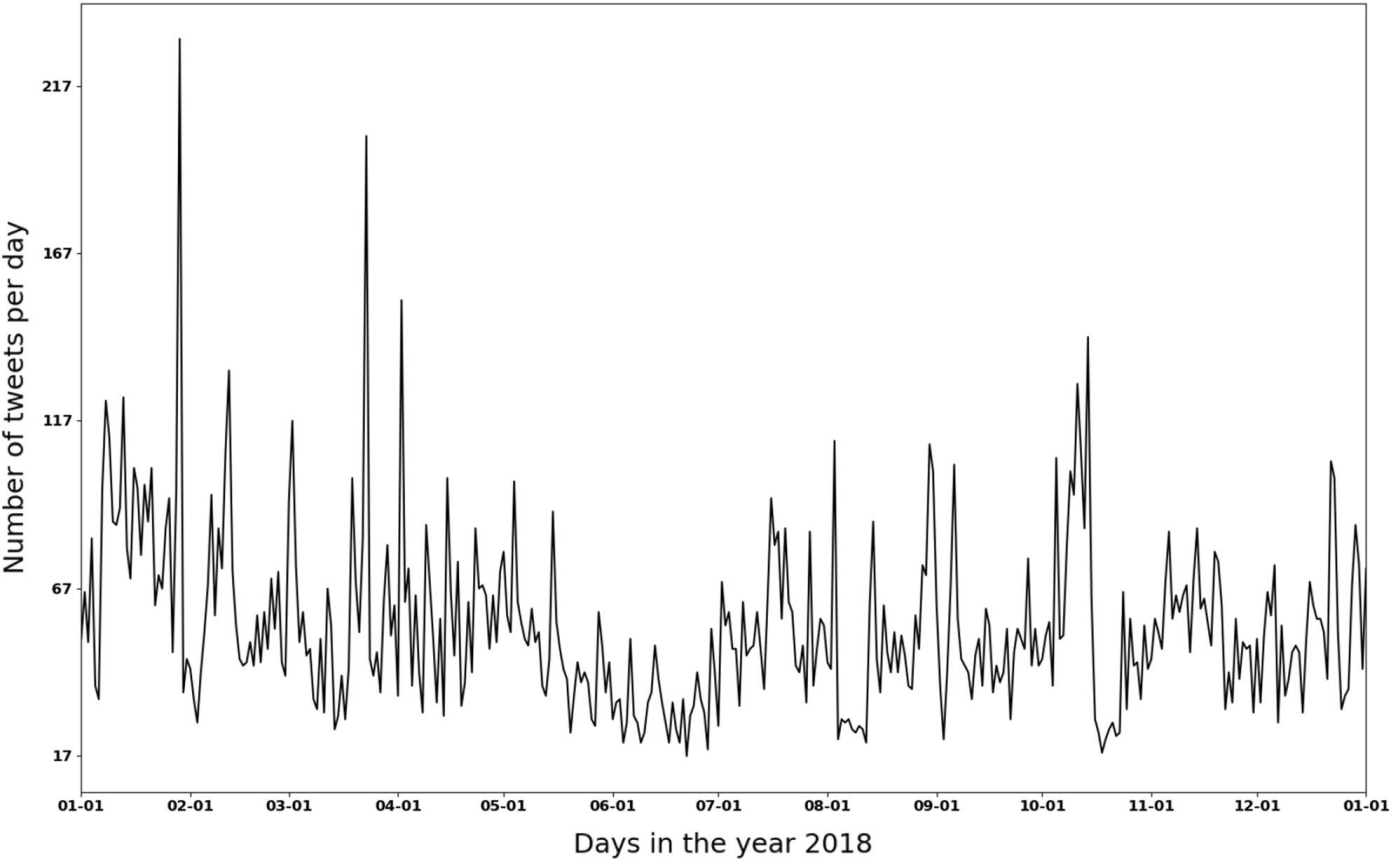
Number of tweets related to foodborne illness incidents in the United States.

In contrast with the results in “Data collection” section that was based on data all over the world, only tweets in the United States will be discussed in this section. *Carmen*, a location inference tool for Twitter data, was used to obtain the location information for each tweet^43^. To note that, the tool utilizes all location-related information, including geolocations and user profiles, to estimate the approximate locations for a tweet. The locations estimated based on user profiles would be less accurate than those from geolocations since the places users mention in their profiles won’t always be the places they post tweets. Due to the fact that there is a lot of missing data in either geolocation along with the posted tweet or the location information in the user’ profiles, the locations identified were accounting for only 1% of all the collected data. Non-US tweets were excluded by removing the tweets whose location was either not identified or in other countries. The remaining tweets were fed into the dual-task BERTweet model to determine if they indicated foodborne illnesses incidences and to extract important entities (e.g., food, location, symptoms). The change of daily volume (number of tweets per day) within the United States domain in 2018 is shown in Fig. 5. It can be seen that the number of tweets per day is significantly smaller than those in Supplementary Figure S2. Some spikes were observed in the days of January–February, March–May, September–October, and in December. However, it is challenging to connect the spikes to a foodborne outbreak due to the noises from sporadic incidents of foodborne illnesses that are not attributing to a foodborne outbreak. On the other hand, multiple foodborne outbreaks can happen spontaneously, which makes the task even more difficult if only the time-series data is provided. More information of relevance to a foodborne outbreak is needed when connecting the trend on Twitter with real-life events.

### Effects of the dual-task model on extracting food entities

The top 20 food entities identified by the dual-task BERTweet model in tweets located in the US and associated with foodborne illnesses included coffee, pizza, chicken, milk, cheese, ice, salad, spices, cream, meat, lettuce, tea, soup, fries, sushi, taco, burger, chocolate, chip, beer, fish, and beef (data not shown). These observations were compared with the foods involved in foodborne outbreaks^44^. It was found that the model not only correctly extracted foods frequently involved in foodborne outbreaks, such as pizza, chicken, milk, cheese, and salad, but also extracted other foods less frequently appeared, such as coffee, ice, tea, chocolate, and chip. It indicates that language structure might be the major factor affecting the entity extraction results, because of whichever elements with similar language structures would be extracted as same type of entities. In addition to specific food terms, the model also extracted general terms such as food, breakfast, drink, dinner, and lunch, due to similar language structure. For example, one might say “I had a pizza yesterday and got sick, vomiting,” or “I had breakfast from McDonald and got food poisoning.” The positions of the words (pizza and breakfast) were the same in the two sentences, and thus can be easily recognized as the same types of entities by the language models. Besides, non-food items such as plastic bags, stomach, eat, half-eaten, and bowl were also extracted, indicating that the model mis-classifies them as food entities. The F1-score of the entity extraction task was 0.61, relatively lower than that of the sentence classification task (0.87).

**Figure 6:**
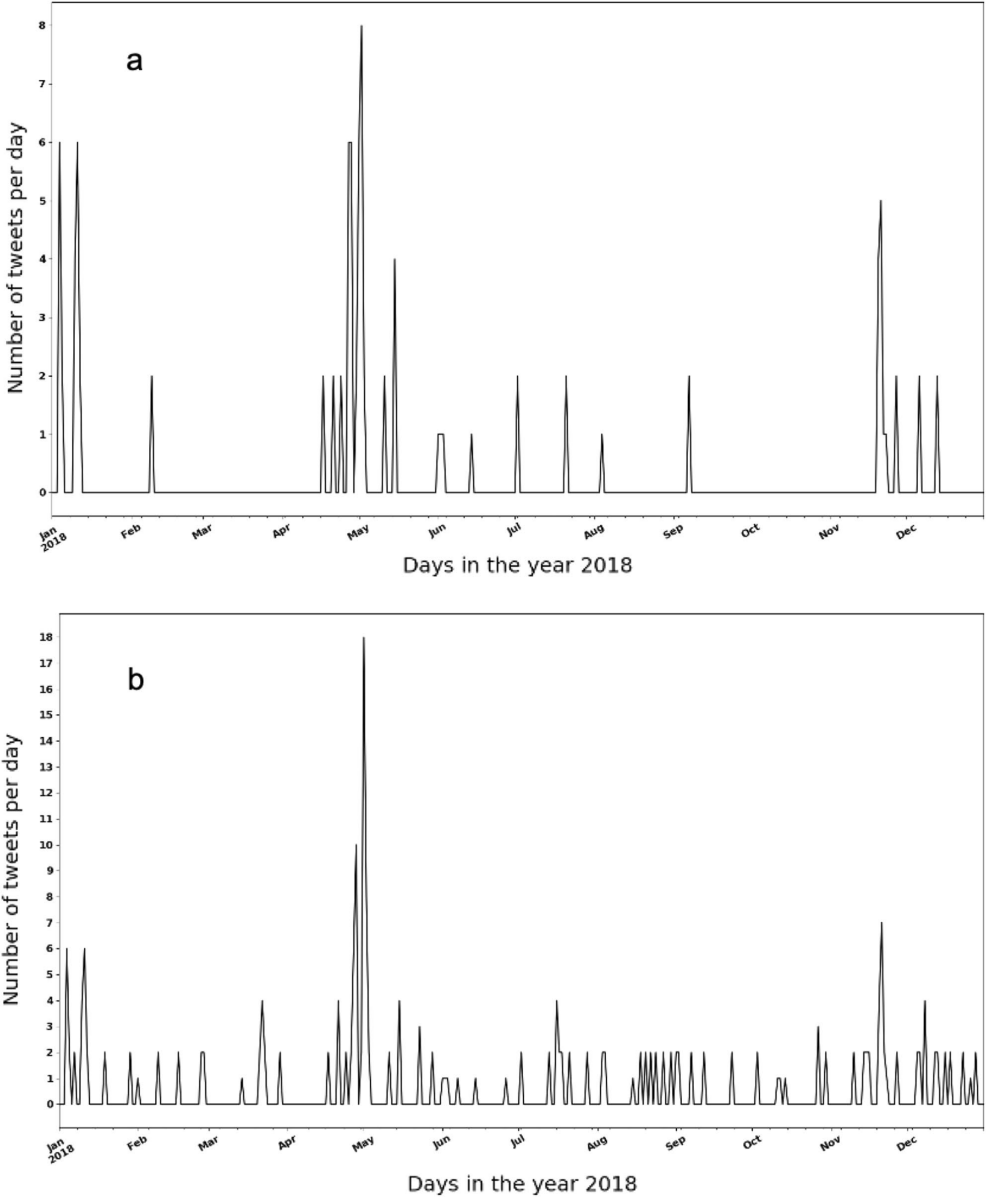
Number of tweets related to foodborne illness incidents in the United States (**a** including “lettuce” in the tweets and **b** including “lettuce” or “salad” or “sandwich” in the tweets).

Named entity extraction for social media data is challenging due to its inherent noisiness such as improper grammatical structures, spelling inconsistencies and numerous informal abbreviations^40^. A variety of methods have been developed to tackle this problem. The BERTweet model was recognized as an effective method to conduct tasks of named entity extraction^25^. The food entities extracted by the model provide extra information for signals of unreported foodborne illnesses, which allows the analysis of foodborne illness incidents associated with specific foods. To connect the volume changes from online data with real-life events, we conducted a case study by selecting specific food entities extracted by the model. As shown in Fig. 6a, changes in the number of tweets indicating foodborne illnesses and mentioning the food entity “lettuce” was observed. It can be seen that there are some spikes in Early January, from April to June, and from November to December. However, the data is relatively small due to the sparsity of location information in the dataset. Two large lettuce-related outbreaks occurred in 2018. One is from March to June, and another is from December to January (2019), indicating the time span of these spikes are consistent with the real-life data. In particular, the romaine lettuce outbreak happened in March 2018 was a serious multi-state outbreak in the United States, leading to 210 illnesses reported by the CDC. We also included more related food items, including “salad” and “sandwich” in which “lettuce” is a common ingredient, to increase the data volume. As shown in Fig. 6b, the spike spanning from April to June becomes significantly more obvious than the other two spikes, with a rapid increase in late April, which is consistent with the romaine lettuce outbreak occurring at the same time.

Social media platforms such as Twitter provide a unique opportunity to monitor food safety related incidents and their spread across time and space in a near real-time fashion. The availability of the *what*, *where* and *when* information about people’s everyday life on the social media websites has proven to be valuable for predicting the flu well before outbreaks have formally been reported by the CDC, and for preventing public health crisis^45^. In the food safety scenario, *what* refers to the content of the tweet describing a potential food safety incident, e.g., the food item and the complaints about it, while *where* and *when* encode the geolocation and the timeframe of the incidents respectively. These properties together form the core entities essential for food safety outbreak monitoring and prevention.

## Conclusions

Social media platform Twitter has been used as a novel data source for identifying unreported foodborne illnesses. A number of language models were developed to classify if a Tweeter post (tweet) indicated a foodborne illness. However, the sporadic signals being captured won’t be able to predict a large-scale outbreak without the availability of critical information related to an outbreak. Therefore, in this work, a dual-task BERTweet model, a derivative of the original BERTweet model, was developed to achieve two goals: (1) classifying if a given tweet was associated with a foodborne illness incidence, and (2) extracting critical entities including food, symptoms and location related to that incidence. The performance of the model in the first task outperformed all previous models with a F1-score of 0.87. The model also showed high precision in extracting specific food entities, using “lettuce” as an example. By narrowing down the dataset to the US domain through location inference, the time-series trend of “sick” tweets related to “lettuce” in 2018 exhibited a similar behavior with the two lettuce-related outbreaks that happened that year. However, the volume became relatively low when focusing on a specific region due to the scarcity of location information. Therefore, the next-step work should focus on how to improve the location inference method so that the dataset could be more precision when connecting to real-life events. Another limitation of this work is data availability. While Twitter provides its API for collecting data for research purposes, the data size available only accounts for about 1% of the whole dataset. As a result, the data for analysis would always be sampled data, which could raise bias concern inherently. More statistical methods should be considered dealing with incomplete sample data when one is trying to connect the observations with a population-level event. Nevertheless, this work develops a model that can provide key elements such as time, location, and food detected from “sick” tweets, which are essential for the development of an early warning method to predict potential foodborne outbreaks in future.

## Supplementary Information

Supplementary Information.

## Data Availability

The datasets collected and analyzed during the current study are available from the corresponding author on reasonable request. In addition, we would like to clarify that the terms of service of Twitter were followed in order to collect the data used in our study. Since the individuals (both the authors of tweets and the annotators at Mturk) involved in our study were not deemed to be human subjects as judged by the Board of Institutional Review Board (IRB), Office for the Protection of Research Subjects (OPRS) at University of Illinois at Urbana-Champaign (IRB#: 21111), and therefore the IRB Board confirmed that the study does not fall under human subjects research and thus it did not require an IRB approval. Through clicking the “Accept” button before starting the tasks on Mturk, the informed consent was received from the participating annotators.
