# Supplementary material for: Crowdsourcing and machine learning approaches for extracting entities indicating potential foodborne outbreaks from social media
Source: Sci Rep. 2021 Nov 4;11:21678. doi: 10.1038/s41598-021-00766-w (PMC8568976; doi:10.1038/s41598-021-00766-w)
Supplement: Supplementary file 1 — Supplementary Information. [file 41598_2021_766_MOESM1_ESM.docx]

**Supplement material files**


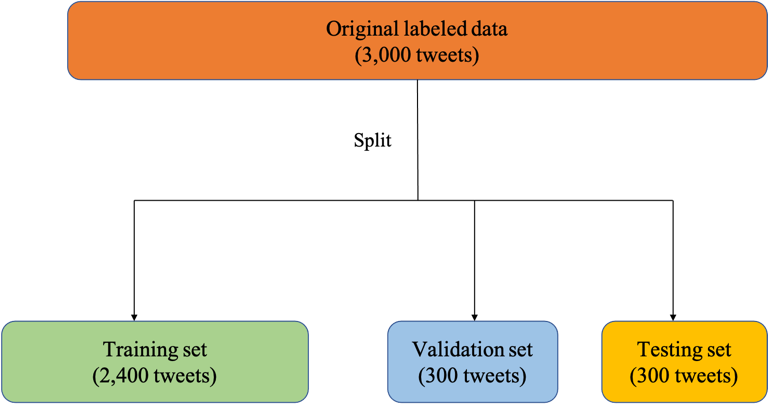


Supplementary Figure S1. Training, validation, and testing set for machine learning.


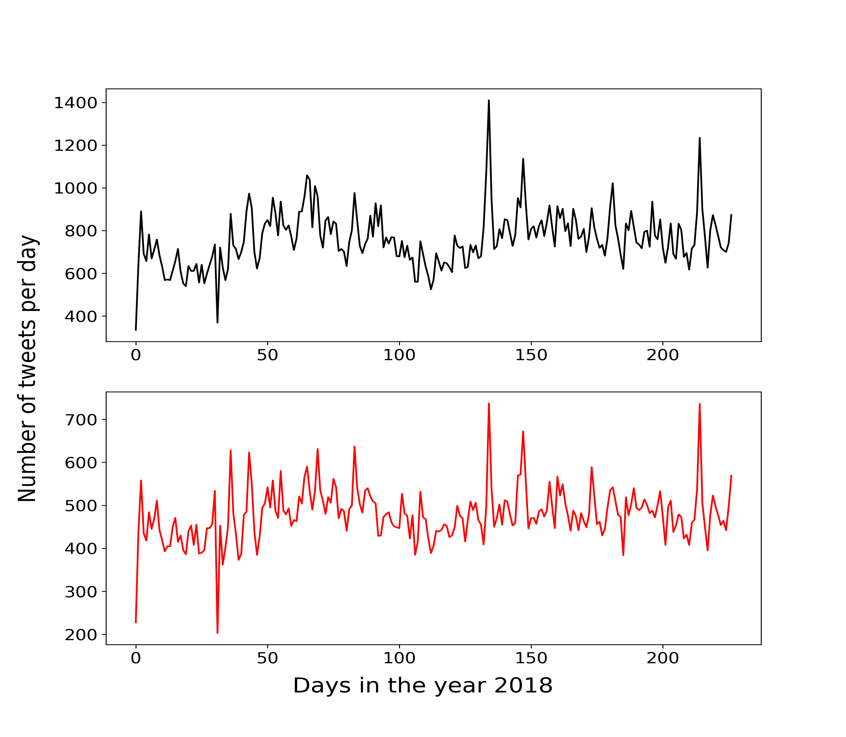


Supplementary Figure S2. Trends of tweets mentioning “food poisoning” in 2018.

Supplementary Table S3. Basic numbers for computing summary statistics in binary classification

|  | True condition | | |
| --- | --- | --- | --- |
| Predicted condition |  | Yes | No |
|  | Yes | True positive (TP) | False positive (FP) |
|  | No | False negative (FN) | True negative (TN) |

Supplementary Table S4. Examples of tweets and the food and symptom entities identified by human taggers.

| No. | Tweets | Food | Symptoms |
| --- | --- | --- | --- |
| 1 | Bowie’s taco salad gave me the worst food poisoning I’ve ever had in my life. We thought I was going to have to go get an IV because I was so dehydrated. | Taco salad | Dehydrated |
| 2 | You know what's NOT great. Eating a salad at the venue you play at only to be so ill from food poisoning you vomit your way home up sydney road. NOT cool #pennyblack. | Salad | Vomit |
| 3 | I got food poisoning from your salad bar. I ate there last night. Was sick all day today, vomiting and diarrhea. Your broccoli salad was bad. So was your lettuce. | Broccoli salad, lettuce | Vomit, diarrhea |
| 4 | I got food poisoning from a bad Subway sandwich. It became a high fever, in which I dreamed a giant sandwich was eating me. I can still remember being squished in the lettuce. So much lettuce. | Sandwich, lettuce | Fever |

Supplementary Table S5. Sentence classification performances: F1-scores.

| Model | Dataset | F1-score |
| --- | --- | --- |
| Baseline individual models |  |  |
| Foodborne Chicago (Harris et al., 2014) | Tweets | NA |
| FINDER (Sadilek et al., 2018) | Tweets | 0.74 |
| FoodSafety SLT (Harries et al., 2018) | Tweets | NA |
| Baseline dual-task models |  |  |
| FoodborneNYC (Effland et al., 2018) | Tweets | 0.84 |
| Dual-task BERTweet model | Tweets | 0.87 |

NA: data not available
